# Management of Patient-Reported Outcome (PRO) Alerts in Clinical Trials: A Cross Sectional Survey.

**DOI:** 10.1371/journal.pone.0144658

**Published:** 2016-01-19

**Authors:** Derek Kyte, Jonathan Ives, Heather Draper, Melanie Calvert

**Affiliations:** Institute of Applied Health Research, University of Birmingham, Birmingham, United Kingdom; Universidad Nacional de la Plata, ARGENTINA

## Abstract

**Background:**

Assessment of patient-reported outcomes (PROs) provides valuable information to inform patient-centered care, but may also reveal ‘PRO alerts’: psychological distress or physical symptoms that may require an immediate response. *Ad-hoc* management of PRO alerts in clinical trials may result in suboptimal patient care or potentially bias trial results. To gain greater understanding of current practice in PRO alert management we conducted a national survey of personnel involved in clinical trials with a PRO endpoint.

**Methods and Findings:**

We conducted a national cross-sectional survey of 767 UK-based research nurses, data managers/coordinators, trial managers and chief/principal investigators involved in clinical trials using PROs. Respondents were self-selected volunteers from a non-randomised sample of eligible individuals recruited via 55 UK Clinical Research Collaboration Registered Clinical Trials Units and 19 Comprehensive Local Research Networks. Questions centred on the proportion of trial personnel encountering alerts, how staff responded to PRO alerts and whether current guidance was deemed sufficient to support research personnel. We undertook descriptive analyses of the quantitative data and directed thematic analysis of free-text comments. 20% of research nurses did not view completed PRO questionnaires and were not in a position to discover alerts, 39–50% of the remaining respondent group participants reported encountering PRO alerts. Of these, 83% of research nurses and 54% of data managers/trial coordinators reported taking action to assist the trial participant, but less than half were able to record the intervention in the trial documentation. Research personnel reported current PRO alert guidance/training was insufficient.

**Conclusions:**

Research personnel are intermittently exposed to PRO alerts. Some intervene to help trial participants, but are not able to record this intervention in the trial documentation, risking co-intervention bias. Other staff do not check PRO information during the trial, meaning alerts may remain undiscovered, or do not respond to alerts if they are inadvertently encountered; both of which may impact on patient safety. Guidance is needed to support PRO alert management that protects the interests of trial participants whilst avoiding potential bias.

## Introduction

Patient-reported outcome (PRO) measures provide a systematic way of assessing patients’ views about their health and well-being [1]. The resulting data are valued by patients [2, 3] and have potential for many uses within healthcare. At an individual level: informing patient choice, facilitating cooperation between healthcare teams to provide tailored individual care and identifying those most in need of intervention [4]. At a macro-level: evaluating treatment safety and effectiveness; and informing prognostic modelling, audit and quality assurance, pay-for-performance initiatives, and health-policy [5–8].

In a routine clinical setting, real-time monitoring of PRO data can allow timely intervention in response to concerning levels of psychological distress or physical symptoms, so-called ‘PRO alerts’ (Box 1) [9]. In clinical trials, however, the management of PRO alerts may vary substantially. Some trials do not review PRO data until the end of the study, which may prevent alert discovery, potentially risking patient safety and legal liability. For those trials that do routinely monitor for PRO alerts, or those where staff are inadvertently alerted, it remains unclear how research personnel minimise the potential for co-intervention bias.

**Box 1. Definitions.**

Patient-Reported Outcome (PRO)–“… any report of the status of a patient’s health condition that comes directly from the patient, without interpretation of the patient’s response by a clinician or anyone else.” [11]

Patient-Reported Outcome Measure (PROM)–A validated paper-based or electronic psychometric questionnaire used to collect PRO data.

PRO Alert–The exposure of data collection staff to PRO data displaying “concerning levels of psychological distress or physical symptoms that may require an immediate response.” [12]

There is little data on how PRO alerts are currently handled in trials [10]. To gain greater understanding of current practice in PRO alert management we conducted a national survey of personnel involved in clinical trials with a PRO endpoint. This report addresses the following questions: What proportion of trial personnel encounter PRO alerts? How do staff respond to PRO alerts and record their intervention? What do research personnel currently receive in the way of guidance, and what guidance would they wish to see distributed in future trials?

## Methods

### Survey development

The survey was developed by investigators with PRO and ethics expertise, informed by our previous qualitative study [10], and refined following pilot testing. Instruments were modified for each participant group (see S1 File). The survey asked: (i) whether respondents had encountered ‘concerning’ PRO trial data in the past; (ii) what, if any actions they had taken in response to this PRO alert, (iii) whether they had been able to record their actions within the trial documentation, (iv) what PRO alert trial training/guidance they had received, (v) what actions they might take in a future trial in response to a PRO alert, and (vi) what PRO alert training/guidance they would like to see provided in future trials. The survey was approved by the West Midlands Research Ethics Committee (ref no 12/wm/0068).

### Survey sample

An anonymised online exploratory cross-sectional national survey of UK research nurses, data managers/coordinators, trial managers and chief and principal investigators (CPIs) involved in clinical trials using either a primary or secondary PRO was undertaken. The survey was anonymised to maximise the number of responses and to encourage respondents to freely discuss potentially controversial aspects surrounding the management of PRO Alerts. Respondents were self-selected volunteers from a non-randomised sample of eligible individuals recruited via 55 UK Clinical Research Collaboration Registered Clinical Trials Units (CRC-RCTUs) and 19 National Institute for Health (NIHR) Comprehensive Local Research Networks (CLRNs). A convenience sample was necessary due to the anonymised nature of the research and the fact that CRC-RCTUs/CLRNs often did not have the resources to maintain up-to-date records regarding the configuration and experience (i.e. previous/current involvement with PROs in trials) of their fluctuating research workforce, meaning a targeted approach was not possible. During recruitment, information about the study was cascaded to all research nurses, data managers/coordinators, trial managers and CPIs at these sites, through their respective research management structures. Interested individuals were asked to visit the online survey site, where they were provided with additional information about the study and the contact details of the lead researcher (DK) if they had further questions. Participants were informed that completion of the online survey constituted research consent and that withdrawal was possible up to the point of survey submission.

### Analysis

Descriptive analysis was used to examine participant characteristics and survey responses. All analysis was conducted using SPSS (version 21, IBM). Free-text comments were analysed by DK using directed content analysis, with an initial coding framework [13] informed by previous qualitative work [10]. Additional codes were developed as the analysis was conducted and the framework was modified as required [13]. JI formally reviewed all coding to enhance trustworthiness, and any coding disagreements were discussed and resolved.

## Results

### Survey respondents

767 participants completed the survey. The characteristics of respondents are shown in Table 1. The survey results are presented in Table 2, and the key findings summarized below alongside illustrative respondent quotations from the free-text comments sections. As ours was an anonymised non-probability sample, a response rate calculation is not appropriate [14]. In addition, neither the UK CRC-CTUs nor the NIHR CLRNs held data regarding the number of staff involved in trials with a primary or secondary PRO, meaning there was no way to determine a denominator. According to the most up-to-date all-staff records provided by the UK CRC-CTU and NIHR CLRN networks, we estimate the total number of UK research nurses, data managers/coordinators, trial managers and CPIs receiving our survey invite, which includes those individuals ineligible for the study, was approximately 1,800; our participants comprised 43% of this figure.

**Table 1:** Characteristics of participants.

Participant Characteristics (total = 767)	No. (%) Research Nurse Participants^a^ (n = 560)	No. (%) Data Manager Participants^a^ (n = 41)	No. (%) Trial Manager Participants^a^ (n = 129)	No. (%) Chief & Principle Investigator Participants^a^ (n = 37)
**Age, years**				
≤25	4 (0.7)	3 (7.9)	4 (3.1)	0 (0)
26–35	95 (17)	14 (36.8)	51 (39.5)	5 (13.5)
36–45	193 (34.5)	10 (26.3)	43 (33.3)	11 (29.7)
46–55	217 (38.8)	8 (21.1)	23 (17.8)	14 (37.8)
≥56	51 (9.1)	3 (7.9)	8 (6.2)	7 (18.9)
**Years in research role**				
<1	51 (9.2)	4 (10.5)	12 (9.3)	0 (0)
1–3	208 (37.3)	13 (34.2)	42 (32.6)	11 (29.7)
4–6	147 (26.4)	7 (18.4)	31 (24)	4 (10.8)
7–9	50 (9)	4 (10.5)	12 (9.3)	5 (13.5)
≥10	101 (18.1)	10 (26.3)	32 (24.8)	17 (45.9)
**Setting of most recent clinical trial collecting PROs**^**b**^				
Primary care	112 (20.7)	15 (39.5)	47 (37.9)	16 (44.4)
Secondary care	428 (79.3)	23 (60.5)	77 (62.1)	20 (56.6)
**Clinical areas covered by most recent clinical trial collecting PROs**^**b**^				
Cardiovascular	69 (16.5)	3 (9.4)	10 (10)	0 (0)
Elderly care	17 (4.1)	2 (6.3)	10 (10)	2 (7.4)
General medicine	39 (9.3)	2 (6.3)	7 (7)	0 (0)
General practice	19 (4.5)	3 (9.4)	23 (23)	9 (33.3)
Neurology	51 (12.2)	1 (3.1)	9 (9)	4 (14.8)
Obstetrics & gynaecology	22 (5.3)	3 (9.4)	7 (7)	2 (7.4)
Oncology	119 (28.5)	15 (46.9)	28 (28)	1 (3.7)
Opthalmology	8 (1.9)	1 (3.1)	4 (4)	7 (25.9)
Orthopaedics	35 (8.4)	1 (3.1)	7 (7)	1 (3.7)
Paediatrics	35 (8.4)	2 (6.3)	9 (9)	6 (22.2)
Respiratory	41 (9.8)	5 (15.6)	8 (8)	3 (11.1)
Rheumatology	47 (11.2)	1 (3.1)	6 (6)	5 (18.5)
**PROs used in trial**^**b**^				
EuroQol EQ-5D	401 (76.1)	25 (67.6)	99 (82.5)	24 (80)
Health Assessment Questionnaire (HAQ)	154 (29.2)	1 (2.7)	4 (3.3)	2 (6.7)
Nottingham Health Profile (NHP)	0 (0)	0 (0)	0 (0)	0 (0)
SF-12 Health Survey or SF-12v2 Health Survey	36 (6.8)	6 (16.2)	22 (18.3)	7 (23.3)
SF-36 Health Survey or SF-36v2 Health Survey	104 (19.7)	5 (13.5)	17 (14.2)	6 (20)
Hospital Anxiety and Depression scale (HAD)	115 (21.8)	4 (10.8)	21 (17.5)	11 (36.7)
Arthritis Impact Measurement Scales (AIMS2)	3 (0.6)	0 (0)	0 (0)	2 (6.7)
EORTC QLQ—C30 (Core Questionnaire)	106 (20.1)	9 (24.3)	18 (15)	0 (0)
Minnesota Living with Heart Failure Questionnaire (MLHF)	9 (1.7)	0 (0)	1 (0.8)	1 (3.3)
Oxford Hip Score (OHS)	9 (1.7)	0 (0)	0 (0)	1 (3.3)
Oxford Knee Score (OKS)	14 (2.7)	1 (2.7)	0 (0)	0 (0)
Roland-Morris Disability Questionnaire (RMDQ)	2 (0.4)	0 (0)	2 (1.7)	4 (13.3)

^a^Columns may not add up to n due to missing values.

^b^Participants could select multiple categories.

**Table 2:** Questionnaire Responses.

Survey Question and Response Options	No. (%) Research Nurse Participants^a^ (n = 560)	No. (%) Data Manager Participants^a^ (n = 41)	No. (%) Trial Manager Participants^a^ (n = 129)	No. (%) Chief & Principle Investigator Participants^a^ (n = 37)
**Have you ever encountered any ‘concerning’ Patient-Reported Outcome information within a trial?**				
Yes	176 (33.8)	14 (46.7)	55 (46.2)	18 (50.0)
No	318 (61.0)	14 (46.7)	62 (52.1)	18 (50.0)
Not applicable/Don't know	27 (5.2)	2 (6.7)	2 (1.7)	0 (0.0)
**Have you ever taken action in response to ‘concerning’ Patient-Reported Outcome information you have encountered within a trial, in order to assist a trial participant?**	100 (19.6)	-	-	-
Yes	145 (82.9)	7 (53.8)	25 (47.2)	15 (83.3)
No	30 (17.1)	6 (46.2)	27 (50.9)	3 (16.7)
***Research nurses who reported they ‘…sent the questionnaire to the data inputting centre without looking at it’ in their last trial collecting PROs***	100 (19.6)	-	-	-
**Were you able to record**^**#**^**/was there a mechanism in place to record**^**~**^ **all action(s) taken in response to the ‘concerning’ Patient-Reported Outcome information, in the trial documentation?**				
Yes	81 (46.0)	4 (30.8)	25 (47.2)	13 (72.2)
No	67 (38.1)	4 (30.8)	27 (50.9)	4 (22.2)
Not applicable	28 (15.9)	5 (38.5)	1 (1.9)	1 (5.6)
**If you**^**#**^**/your data collection staff**^**~**^ **were to encounter ‘concerning’ Patient-Reported Outcome information in a future trial, for example, evidence of anxiety or depression, which of the following would you do**^**#**^**/ expect them to do?** ^**~**^				
I would not intervene^#^/not to intervene^~^, it is the responsibility of the trial participant's GP and regular healthcare team to monitor and deal with quality of life related disorders such as anxiety and depression, not the trial staff.	13 (2.6)^b^	11 (42.3)^b^	27 (24.3)^b^	4 (12.1)^b^
Discuss the findings a their line manager in the trial, or with the PI.	389 (77.5)^b^	14 (53.8)^b^	88 (79.3)^b^	27 (81.8)^b^
Discuss the findings with a colleague.	111 (22.1)^b^	1 (3.8)^b^	9 (8.1)^b^	7 (21.2)^b^
Discuss the findings with the participant.	335 (66.7)^b^	-	27 (24.3)^b^	17 (51.5)^b^
Using discretion, arrange an appointment with the patient's GP or other appropriate healthcare professional.	119 (23.7)^b^	-	19 (17.1)^b^	10 (30.3)^b^
I would not intervene, there is nothing I could do.	-	2 (7.7)^b^	-	-
I would discuss the findings with the participants research nurse.	-	11 (42.3)^b^	-	-
**What particular information on Quality of Life/Patient-Reported Outcome measurement was given to the data collection staff?**				
How to deal with Quality of Life/Patient-Reported Outcome information that raises concern for the wellbeing of the trial participant (e.g. a questionnaire indicating severe anxiety or depression).	-	-	31 (38.3)	22 (75.9)
**There is usually specific guidance on dealing with ‘concerning’ Patient-Reported Outcome information contained in trial protocols.**				
Yes (agree)	65 (12.7)	8 (28.6)	-	-
No (disagree)	265 (52.0)	14 (50.0)		
Unsure	180 (35.3)	6 (21.4)		
**I have had trial training on what to do if I encounter 'concerning' Patient-Reported Outcome information.**				
Yes (agree)	59 (11.6)	6 (21.4)	-	-
No (disagree)	417 (81.9)	21 (75.0)		
Unsure	33 (6.5)	1 (3.6)		
**I feel confident about dealing with ‘concerning’ Patient-Reported Outcome trial information.**				
Yes (agree)	279 (54.5)	11 (39.3)	-	-
No (disagree)	97 (18.9)	8 (28.6)		
Unsure	136 (26.6)	9 (32.1)		
**There should be specific protocol content and trial training on how to deal with ‘concerning’ Patient-Reported Outcome information, in trials employing such outcomes.**				
Strongly Agree	140 (36.5)	7 (25.0)	27 (47.2)	14 (38.9)
Agree	283 (54.1)	11 (39.3)	70 (60.9)	17 (47.2)
No opinion	57 (6.4)	5 (17.9)	12 (10.4)	3 (8.3)
Disagree	20 (2.8)	4 (14.3)	5 (4.3)	2 (5.6)
Strongly Disagree	1 (0.2)	1 (3.6)	1 (0.9)	0 (0.0)
**Thinking about the future. Where should guidance on when/how to deal with 'concerning' Quality of Life/Patient-Reported Outcome information be provided?**				
Trial Protocol	270 (55.3)	10 (37.0)	49 (43.8)	15 (46.9)
Trial Training	407 (83.4)	20 (74.1)	95 (84.8)	27 (84.4)
Standard Operating Procedure	283 (58.0)	13 (48.1)	77 (68.8)	28 (87.5)

^a^Columns may not add up to n due to missing values.

^b^Participants could select multiple categories.

^#^Indicates question to research nurses and data managers.

^~^indicates question to trial managers and CPIs.

### PRO alert management

Survey respondents were asked if they had encountered ‘concerning’ PRO data within a trial and, if so, whether they had taken any action in response to it. 34% of research nurses, 47% of data managers/coordinators, 46% of trial managers and 50% of CPIs reported encountering an alert. Of these, 83% research nurses, 54% data managers/coordinators, 47% trial managers and 83% of CPIs reported taking action aimed at assisting the trial participant. It is notable that 20% of research nurses reported sending their PRO questionnaires directly to the data inputting centre without looking at it and were therefore not in a position to discover a PRO alert.

There were 144 free-text comments in this section, which indicated variation in the factors reported to trigger a PRO alert for different individuals. The majority cited signs of depression and/or suicidal ideation as the initial trigger:

*‘… patient who repeatedly said she was fine in clinic but scored high for depression… consultant and I discussed scores with patient*, *referred to hospital psychologist… GP [general practitioner] prescribed antidepressants*.*’ [Research Nurse]*

*‘Patient reported suicidal feelings… reported to co-investigator and PI [principal investigator]*.*’ [Research Nurse]*

Other staff reported responding to signs of ‘low mood’ or reduced mental-wellbeing:

*‘Expression of overwhelming not coping or sadness–use[d] the form completion as an opening to start discussion about the fact there may be an issue and refer to those who can help…’ [Research Nurse]*

Extreme PRO questionnaire scores were a potential alert trigger for some:

*‘If HAD [Hospital Anxiety and Depression scale] scores were over 11 then we reported them to the GP with the participant's consent*. *We also had a psychologist attached to the cardiac rehab team who would take referrals with the participant’s consent*.*’[Research Nurse]*

*‘…abnormal HADs scores are reported to the participant's GP*. *Trial nurses/Doctors have also been alerted if something needs following up’ [Data Manager]*

*‘a 12 year old scored 30 on a quality of life health questionnaire*, *I informed her consultant who was also the study PI and her specialist nurse*.*’ [Research Nurse]*

Other comments suggested staff also responded to PRO reports of reduced physical wellbeing (e.g. pain, discomfort, vomiting):

*‘Pain score was severe therefore I reported it to the relevant clinician*. *I then ensured that this had been acted upon*.*’ [Research Nurse]*

Free-text comments outlining the actions taken in response to an alert also suggested variability amongst respondents. Most indicated that staff tended to refer to a clinically qualified professional external to the trial team, sometimes with the participant’s prior permission:

*‘… patient may express concerns re their analgesia*, *deteriorating symptoms*, *need for help with psycho-social issues*. *I make an entry into the notes and alert the healthcare professional responsible for the participant's care via email*.*’ [Research Nurse]*

*‘During a mental health trial I reported concerns to a GP with the participant's permission due to the nature of answers given*.*’ [Research Nurse]*

Other comments suggested staff discussed alert findings directly with the trial participant, with some respondents commenting that they advised the participant to seek medical advice independently:

*‘Discussed the issue with participant to see if any further action… required’ [Research Nurse]*

*‘Advised them to make an appointment to see their GP’ [Research Nurse]*

A small number of free-text comments suggested staff informed members of the trial management team:

*‘Higher than previously reported depression score*. *I fed the information back to the PI once I had chatted to the patient to establish that they had answered honestly and accurately’ [Research Nurse]*

*‘Spoke with the PI immediately in order to ascertain whether an urgent psychological review was required*.*’ [Research Nurse]*

Finally, two comments suggested there were formal trial procedures in place to handle PRO alerts:

*‘We wrote it in the trial protocol that we would contact the patients clinician if they scored highly in the HADS questionnaire*.*’ [Trial Manager]*

*‘Official process (explained in PIS [patient information sheet]) for alerting investigators if participants responses on [questionnaire] …suggested suicidal ideation*.*’[Trial Manager]*

### Alert documentation

Respondents involved in trial management (trial managers and CPIs) were asked, with regard to the most recent trial in which they were involved, if there was a mechanism in place to record actions taken in response to a PRO alert. 72% of CPIs and 47% of trial managers reported that such a mechanism was present. In contrast, and apparently inconsistently, 46% of research nurses and 31% of data manager/coordinators reported that they had been able to record their PRO alert responses in the trial documentation.

There were 46 free-text comments in this section. The majority of research nurse comments suggested that responses to PRO alerts were recorded in the participant’s general medical notes:

*‘This was not something that the trial documentation was designed for so concerns and actions would have been documented in patient's notes*.*’ [Research Nurse]*

However, some trial managers reported that they were recorded in the trial documentation as a file note or a specific database entry:

*‘Comments entered on database*, *copy of questionnaire kept in file (as usual)*, *and documentation of telephone calls with patient and GP*, *and copy of fax to GP all retained in file*.*’ [Trial Manager]*

A small number of comments from both groups suggested responses to alerts would be detailed in a ‘formal risk report’, Adverse Event (AE) or Serious Adverse Event (SAE):

*‘Risk reports always have to be completed and sent to the GP’ [Trial Manager]*

*‘…it would be noted as an AE and recorded accordingly’ [Research Nurse]*

### Future actions: Trial management staff

CPIs and trial managers were asked how they would expect their data collection staff to manage PRO alerts in future trials. 82% of CPIs and 79% of trial managers suggested that staff should discuss the findings with their line manager/principal investigator, or with the trial participant (52% and 24% respectively). 30% of CPIs and 17% of trial managers expected data collection staff to use their discretion and arrange an appointment with the participant’s general practitioner (GP) or other appropriate healthcare professional. A minority, 12% of CPIs and 24% of trial managers, felt staff should not intervene, favouring leaving the participant's GP and clinical team to monitor and deal with emerging health issues. Finally, 21% of CPIs and 8% of trial managers thought that data collection staff should discuss the alert with a colleague.

### Future actions: Front-line trial personnel

Research nurses and data managers/coordinators were asked how they would respond to a PRO alert in a future trial. A majority of both groups, 78% of research nurses and 54% of data managers/coordinators, indicated they would discuss the concerns with their line manager or the lead investigator. 67% of research nurses reported they would discuss information that concerned them with the trial participant him/herself and 24% that they would use their discretion and arrange an appointment with the participant’s GP if necessary. A lower proportion, 22% of research nurses and 4% of data managers/coordinators, reported that they would discuss alerts with a colleague. Just 3% of research nurses indicated that they would not intervene if they encountered ‘concerning’ PRO data. A greater proportion of trial managers/coordinators indicated they would refrain from intervening, either because they felt the participant’s GP should manage health issues (42%) or because they felt there was nothing they could do to help (8%).

### Trial level guidance

76% of CPIs and 38% of trial managers reported that alert guidance was included either in the protocol or in training/standard operating procedures (SOPs) provided during the most recent trial in which they had been involved. In contrast, 13% research nurses and 29% data managers/coordinators reported the presence of such guidance. Similarly, only 12% of research nurses and 21% of data managers/coordinators reported receiving trial training incorporating PRO alert guidance.

84% of research nurses, 64% of data managers/coordinators, 84% of trial managers and 86% of CPIs agreed or strongly agreed that there should be specific protocol content and/or trial training on how to deal with ‘concerning’ PRO information in trials with PRO outcomes. Survey respondents were asked to indicate a preference about where such information should appear: the trial protocol, in trial training, or in supporting trial documentation. The majority of all respondent groups selected the option for guidance to be included in trial training (research nurses, 74%; data managers/coordinators, 83%; trial managers 85%; CPIs, 84%). A majority of data managers/coordinators, trial managers and CPIs also opted for the inclusion of guidance in supporting documentation (58%, 69% and 88% respectively). Data managers/coordinators were the only group with a majority selecting inclusion of guidance in the trial protocol (55%).

## Discussion

Our main findings suggest a broad range of staff encounter PRO alerts, but that management of these alerts is inconsistent. This may lead to suboptimal patient care, where no response is forthcoming, or potentially biased trial results when research personnel intervene to aid the trial participant but do not consistently record their interventions in the trial documentation. Furthermore, the data suggest there may be a lack of PRO alert guidance for front-line data collection staff, both in trial protocols and training. This may in-part explain the wide variation seen in our sample with regard to the factors that trigger a PRO alert for different individuals, the nature of their subsequent response, and the way in which the response is recorded in the trial, if at all.

A minority of respondents indicated they would not respond to a PRO alert, feeling the participant’s regular healthcare team should manage their patient’s care. It is not clear, however, how potential participant distress captured by a trial PRO would be discovered and managed in routine healthcare practice, as not all providers ask their patients to routinely complete PROs for clinical monitoring purposes or to guide ‘real-time’ clinical decisions. Moreover, unless they are given information to the contrary, trial participants may assume that their PRO responses will be followed-up by the trial team and therefore may not think it necessary to contact their healthcare team for help. In these circumstances, if the trial team does not monitor and respond to a PRO alert, the participant may not be offered appropriate care, potentially leading to unnecessary suffering and poorer outcomes. Failing to respond to a PRO alert arguably represents an abdication of responsibility by the study team, who are ethically and legally obliged to place the safety and wellbeing of research participants ahead of the interests of the trial [15–20]. In addition, participants experiencing poor outcomes are more likely to drop out of trials, increasing rates of missing data and potentially affecting the integrity of trial results [21].

There is a potential risk, however, that research personnel who do respond to PRO alerts influence the primary outcome of a trial by unwittingly introducing ‘co-intervention bias’. This is bias caused by “any intervention other than the experimental manoeuvre that alters the frequency of a trial’s outcome of interest” [22]. For instance, in some trials, higher levels of toxicity or side effects experienced by participants in one study arm may lead to more co-interventions, which, if not recorded, may result in an overestimation of the benefits (including cost-effectiveness) of treatment delivered in that arm of the trial. It is not possible to determine if such co-intervention bias was present in trials involving our respondents. However, as our data suggest not all staff responding to a PRO alert recorded their intervention, it remains a possibility. Steps should be taken to ensure that all co-interventions are recorded in a consistent manner and appropriately monitored so they are available for analysis where appropriate.

Despite many CPI respondents reporting that adequate PRO alert guidance was provided in trial protocols and training, the majority of trial managers, data managers/coordinators and research nurses felt guidance was lacking. The responses of these latter groups concur with the results of our recent study evaluating the PRO-specific content of trial protocols [23], where only 11% of protocols were found to include PRO alert guidance. More than three-fifths of all survey respondents reported wanting specific protocol content and/or trial training on how to deal with PRO alerts in future trials.

Our findings suggest that trial management groups should acknowledge the potential for (and the implications of) PRO alerts in the design phase of the study and should produce appropriate management instructions, made available to all data collection staff, where alerts are a possibility. Three viable methods for monitoring and managing PRO alerts in trials have been reported in the literature [9]. First, participants could be provided with a 2-part disclaimer during enrolment clearly stating that PRO data collected during the trial will not be used to inform clinical management and explaining the route via which participants in need should independently seek assistance from their existing health care provider. Second, retaining the disclaimer outlined above, the trial management group may also wish to provide a study-based support mechanism, for example a 24-hour telephone helpline. Third, the trial may choose to actively monitor for PRO alerts, managing potential patient distress according to a pre-specified action plan communicated to all staff deemed responsible for this aspect of the study. Each of these approaches has its own advantages and disadvantages [9]. It is likely that each trial team will need to carefully consider the risk profile of their study, alongside its staffing structure and resource level, before deciding on the optimal alert management procedures that should be in place.

Formal guidance on how to manage PRO alerts is generally lacking in the literature [24]. This may explain the absence of agreement between our survey groups regarding the most appropriate way to manage PRO alerts in future trials. There is a need to develop consensus guidelines on PRO alert management in clinical trials, aimed at protecting participants, supporting appropriate PRO trial design and outlining the key considerations for researchers. Furthermore, if it is not clear to the trial teams how PRO alerts are to be monitored and responded to, it is reasonable to assume that participants will not understand how their PRO data will be used in the study, including who will access the data and for what purpose. This has the potential to undermine the validity of informed consent if a participant’s expectations are not congruent with actual practice.

Finally, whilst the focus of this research was on the management of PROs in clinical trials, the issue of PRO alerts is also pertinent to PRO use in routine clinical practice. As PROs are increasingly used in clinic to facilitate tailored individual care, and are further integrated into Electronic Health Records (EHRs) and in big data for macro-level health care decision-making [14], it is important that clinicians and providers incorporate appropriate management of PRO alerts into their procedures to protect patient safety.

Our study has some limitations. Respondents were self-selecting and may be more likely to include those with an interest in PROs, whose data could represent that of the most knowledgeable trial personnel. This should be taken into account when interpreting the results of the study. The levels of PRO Alerts reported in this study may be an underestimate; in the absence of pre-specified formal systematic checking of PRO data for alerts, those described in the survey may simply reflect chance encounters. The large research nurse sample size in this study enhances generalisability of the results in this group. However, further research is needed to establish the external validity of the results for the other respondent groups owing to their lower sample sizes. Respondent free-text comments should also be interpreted with caution as not all participants reported their actions or viewpoint. As ours was an anonymised non-probability sample we were unable to determine either the level, or characteristics, of non-responders, meaning our results may be affected by non-response bias. In addition, it was not possible to link respondents together on a particular study. It is therefore important that further work is conducted to establish if the PRO alert management and co-intervention variability seen in this survey may be present in a single trial.

## Conclusions

PRO data collected in clinical trials can give rise to ‘PRO alerts’. Some staff do not check PRO information during the trial, meaning alerts may remain undiscovered, or do not respond to alerts if they are inadvertently encountered. Failure to monitor and react may impact on patient welfare and safety and raises issues around legal liability. When PRO alerts *are* acted upon, staff intervene to aid participants, but some may not be able record the co-intervention in the trial documentation, potentially leading to co-intervention bias. Trials should have an *a priori* plan in place to deal with PRO alerts, aimed at ensuring that participants in need are managed appropriately, whilst also facilitating unbiased PRO data collection and analysis.

## Supporting Information

S1 File Survey Instruments. (PDF)

S2 File Survey Dataset. (XLSX)

## Abbreviations

PROs: patient-reported outcomes
PROMs: patient-reported outcome measures
SOPs: standard operating procedures
CPIs: chief and principal investigators
