# Supplementary material for: Management of Patient-Reported Outcome (PRO) Alerts in Clinical Trials: A Cross Sectional Survey
Source: PLoS One. 2016 Jan 19;11(1):e0144658. doi: 10.1371/journal.pone.0144658 (PMC4718453; doi:10.1371/journal.pone.0144658)
Supplement: S1 File — (PDF) [file pone.0144658.s001.pdf]

Appendix 1

Survey instruments and pilot survey details

National Research Nurse Survey

Introduction

Thank you for your interest in this research

What is the purpose of this study?

The aim of this national survey is to gather information on how research nurses deal with quality of life and other patient-reported outcomes in clinical trials. We will donate £2 to Cancer Research UK for each completed survey we receive.

Who is doing this research?

This research is being conducted by the 'Patient-Reported Outcomes Research Group' based in Primary Care Clinical Sciences at the University of Birmingham. The study forms part of a PhD being undertaken by Derek Kyte MSc, supervised by Dr Melanie Calvert PhD, Professor Heather Draper PhD and Dr Jonathan Ives PhD. The West Midlands Research Ethics Committee have favorably reviewed the study (Reference number: 12/WM/0068).

How long will it take?

The questionnaire has 19 questions, expected completion time is 10-15 minutes. During the pilot phase, the average completion time was 11 minutes.

How will my data be protected?

All of the data collected from you will be kept anonymous. You will not be asked for any personal information, such as your name, date of birth or contact details, any other potentially identifying data will be kept confidential or anonymised. We will ask you about your work experience, but this information will be analysed at a group level and individual details will not be shared. The results of the questionnaire and any reports derived from it will be securely stored on the computer systems in Primary Care Clinical Sciences at the University of Birmingham for the duration of 10 years. After this period they will be deleted so that they cannot be recovered. Reports derived from the questionnaire results will be published in peer reviewed scientific journals, all data will be anonymous.

Once I agree to take part, can I change my mind?

You can exit the questionnaire at any point prior to submission and your answers will not be analysed. Once you have submitted the completed questionnaire you will not be able to withdraw as there is no way we can retrieve your anonymised answers.

Who can I contact to ask any questions?

Mr Derek Kyte
Phone: 0121 4158502, Email: d.g.kyte@bham.ac.uk

Who can I contact if I wish to make a complaint?

Dr Melanie Calvert
Phone: 0121 4148595, Email: m.calvert@bham.ac.uk

Please note: by advancing to the next page, you are consenting to take part in the study. Anonymity and confidentially will be ensured.

Explanation

National Research Nurse Survey

DEFINITIONS OF TERMS USED IN THE SURVEY

Patient-reported outcome measures

Patient-Reported Outcome measures ask the patient a series of questions in order to gauge their views on their own health or care, i.e. an outcome directly reported by the patient.

Quality of Life

A Quality of Life measure is a Patient-Reported Outcome which is designed to evaluate the way in which physical, emotional and social well-being are affected by a disease or its treatment.

SECTION 1

The next section asks questions about your experience of the **last** clinical trial you worked on that used a quality of life or other patent-reported outcome measure. It does not matter is this was a primary or secondary outcome in the trial.

SECTION 1 - Last trial

1. First, some questions about yourself. How long have you been qualified as a nurse?

- ○ Less than 1 year
- ○ 1-3 years
- ○ 4-6 years
- ○ 7-9 years
- ○ 10 years or more

2. How long have you worked as a research nurse in total?

- ○ Less than 1 year
- ○ 1-3 years
- ○ 4-6 years
- ○ 7-9 years
- ○ 10 years or more

3. Which of the following age groups do you belong to?

- 25 or younger
- 26-35
- 36-45
- 46-55
- 56 or older

4. Thinking about the last trial you worked on that used a Quality of Life or other Patient-Reported Outcome measure.

Were you employed as a research nurse in primary care or secondary care?

- Primary Care
- Secondary Care

Other (please specify)

5. Which of the following clinical areas did the trial cover? PLEASE TICK ALL THAT APPLY

- General Practice
- Orthopaedics
- General Medicine
- Rheumatology
- Cardiovascular
- Oncology
- Elderly Care
- Respiratory
- Opthalmology
- Obstetrics & Gynaecology
- Paediatrics
- Neurology

Other(s) (please specify)

6. Which of the following Patient-Reported Outcome Measures did the trial use? PLEASE TICK ALL THAT APPLY

- ☐ Euroqol EQ-5D
- ☐ Health Assessment Questionnaire (HAQ)
- ☐ Nottingham Health Profile (NHP)
- ☐ SF-12® Health Survey or SF-12v2™ Health Survey
- ☐ SF-36® Health Survey or SF-36v2™ Health Survey
- ☐ Hospital Anxiety and Depression scale (HAD)
- ☐ Arthritis Impact Measurement Scales (AIMS2)
- ☐ EORTC QLQ - C30 (Core Questionnaire)
- ☐ Minnesota Living with Heart Failure © Questionnaire (MLHF)
- ☐ Oxford Hip Score (OHS)
- ☐ Oxford Knee Score (OKS)
- ☐ Roland-Morris Disability Questionnaire (RMDQ)
- ☐ Don't know
- ☐ Can't remember

Other(s) (please specify)

SECTION 1 - Last trial

7. Thinking about the last trial you worked on that used a Quality of Life or other Patient-Reported Outcome measure.

What assistance did you give to the trial participants during the completion of the questionnaire? PLEASE TICK ALL THAT APPLY

- ☐ I read the questions out to the participants.
- ☐ I helped participants to understand the questions.
- ☐ The participants gave me the answers and I filled in the questionnaire.
- ☐ I gave no assistance, the participants filled in their questionnaires independently.

Other (please specify)

8. During this trial, if the participant had to complete the Quality of Life or other Patient-Reported Outcome Measure questionnaire in clinic, when did they do so?

- Always before their Consultant/Doctor appointment.
- Always after their Consultant/Doctor appointment.
- Variable, sometimes before and sometimes after their Consultant/Doctor appointment.
- Not applicable.

Other (please specify)

9. During this trial, which of the following did you do after trial participants had completed their Quality of Life/Patient-Reported Outcome questionnaires? PLEASE TICK ALL THAT APPLY

- I sent the questionnaire to the data inputting centre without looking at it.
- I looked at the completed questionnaire to see if the participant had missed out any questions.
- If I discovered missing items, I prompted participants to complete them.
- I looked at the completed questionnaire to see if there were any scoring errors (e.g. 2 options selected instead of 1, scoring the wrong way round etc).
- If I suspected a scoring error, I prompted participants to look again at some questions, to ensure they had understood them correctly.

You may expand upon your answers here (optional)

SECTION 1 - Last trial

10. Thinking about the <u>last</u> trial you worked on that used a Quality of Life or other Patient-Reported Outcome measure.

Please read the following statements. In each case, please answer 'yes', 'no', or 'not applicable'.

	Yes	No	Not applicable
The trial **protocol** included information about Quality of Life/Patient-Reported Outcome measurement.	◌	◌	◌
I felt the trial **protocol** content covering Quality of Life/Patient-Reported Outcome measurement was adequate for my needs.	◌	◌	◌
I received trial **training** that included information on Quality of Life/Patient-Reported Outcome measurement.	◌	◌	◌
I felt the trial **training** I received on Quality of Life/Patient-Reported Outcome measurement was adequate for my needs.	◌	◌	◌

You may expand upon your answers here (optional)

11. Thinking about the last trial you worked on that used a Quality of Life or other Patient-Reported Outcome Measure.

Please read the following statements. In each case, please answer 'yes' or 'no'.

	Yes	No
It was explained to me why the Quality of Life/Patient-Reported Outcome Measure data was being collected in the trial.	◯	◯
I was confident I could explain to trial participants why the Quality of Life/Patient-Reported Outcome Measure data was being collected in the trial.	◯	◯
It was explained to me why each of the questions in the Quality of Life/Patient-Reported Outcome Measure were included, i.e. how each was of relevance to the trial.	◯	◯
I was confident I could explain to trial participants why each of the questions in the Quality of Life/Patient-Reported Outcome Measure had been included, i.e. how each was of relevance to the trial.	◯	◯

You may expand upon your answers here (optional)

SECTION 2 - General thoughts

SECTION 2

This section will ask about your **general** thoughts about working with Quality of Life/Patient-Reported Outcome Measures in trials.

SECTION 2 - General thoughts

12. Thinking about your general experience of Quality of Life/Patient-Reported Outcome measurement in trials.

Please read the following statements. In each case, tick one option: 'always', 'often', 'sometimes', 'never'.

	Always	Often	Sometimes	Never
I **feel bad** giving the trial participant a Quality of Life/Patient-Reported Outcome questionnaire to fill in because the content might upset them.	○	○	○	○
I **feel upset** during the administration of a Quality of Life/Patient-Reported Outcome questionnaire, or when I have read the participant's answers.	○	○	○	○
I **feel a tension** between my role as a nurse and my role as a researcher when dealing with Quality of Life/Patient-Reported Outcome measurement within a trial.	○	○	○	○

You may expand upon your answers here (optional)

SECTION 2 - General thoughts

13. Some research nurses we have spoken to have reported encountering Quality of Life/Patient-Reported Outcome questionnaires containing answers which raise concern for the wellbeing of the trial participant in some way.

This information has been termed: 'concerning' Patient-Reported Outcome information.

In these reports, 'concerning' Patient-Reported Outcome information may simply have been particularly extreme questionnaire scores, or sometimes a participant might have written additional information on the questionnaire which raised concern (or attached a letter); finally, some nurses reported becoming concerned by things that the trial participant said to them either during, or after, the completion of the questionnaire.

Have you ever encountered any 'concerning' Patient-Reported Outcome information within a trial?

- ○ No
- ○ Yes
- ○ Don't know

SECTION 2 - General thoughts

14. Have you ever taken action in response to 'concerning' Patient-Reported Outcome information you have encountered within a trial, in order to assist a trial participant?

- ○ No
- ○ Yes

Please provide details here of **what caused you to become concerned** and **what actions were taken** (optional)

15. Were you able to record all action(s) taken in response to the 'concerning' Patient-Reported Outcome information, in the trial documentation?

- ○ No
- ○ Yes
- ○ Not applicable

You may expand upon your answer here (optional)

SECTION 2 - General thoughts

16. If you were to encounter 'concerning' Patient-Reported Outcome information in a future trial, for example, evidence of anxiety or depression, which of the following might you consider doing? PLEASE TICK ALL THAT APPLY

- ☐ I would not intervene, it is the responsibility of the trial participant's GP and regular healthcare team to monitor and deal with quality of life related disorders such as anxiety and depression, not the trial staff.
- ☐ I would discuss the findings with my line manager in the trial, or with the PI.
- ☐ I would discuss the findings with a colleague.
- ☐ I would discuss the findings with the participant.
- ☐ Using my discretion, I would arrange an appointment with the patient's GP or other appropriate healthcare professional.

Other (please specify)

National Research Nurse Survey

17. Please read the following statements. In each case, please answer 'yes', 'no', or 'unsure'

	Yes	No	Unsure
There is usually specific guidance on dealing with ‘concerning’ Patient-Reported Outcome information contained in **trial protocols**.	◌	◌	◌
I have usually had **trial training** on what to do if I encounter 'concerning' Patient-Reported Outcome information.	◌	◌	◌
I feel **confident** about dealing with ‘concerning’ Patient-Reported Outcome trial information.	◌	◌	◌

You may expand upon your answers here (optional)

SECTION 3 - The future

SECTION 3

This final section will ask about the changes you would like to see regarding Quality of Life/Patient-Reported Outcome measurement in **future trials**.

SECTION 3 - The future

18. Thinking about the future.

Please read the following statements. In each case, please indicate whether you 'strongly agree', 'agree', 'have no opinion', 'disagree' or 'strongly disagree' with the statement.

	Strongly Agree	Agree	No Opinion	Disagree	Strongly Disagree
There should be more **protocol content and trial training** covering Quality of Life/Patient-Reported Outcome measurement, in trials employing such outcomes.	◯	◯	◯	◯	◯
There should be more Quality of Life/Patient-Reported Outcome measurement guidance contained within **other trial documentation**, such as site manuals or standard operating procedures, in trials employing such outcomes.	◯	◯	◯	◯	◯
There should be specific **protocol content and trial training** on how to deal with **'concerning'** Patient-Reported Outcome information, in trials employing such outcomes.	◯	◯	◯	◯	◯

You may expand upon your answers here (optional)

19. Thinking about the future.

What particular Quality of Life/Patient-Reported Outcome guidance should be included the trial protocol, what should be included in trial training, and what should be included in a standard operating procedure? PLEASE TICK ALL THAT APPLY

	Trial Protocol	Trial Training	Standard Operating Procedure
Purpose/Importance of Quality of Life/Patient-Reported Outcome data in trial.	☐	☐	☐
How to administer the questionnaire.	☐	☐	☐
When to administer the questionnaire.	☐	☐	☐
When/how to deal with 'concerning' Quality of Life/Patient-Reported Outcome information.	☐	☐	☐
What to do if participants write additional information on their questionnaires (or attach a letter).	☐	☐	☐
Ethical issues associated with Quality of Life/Patient-Reported Outcome use.	☐	☐	☐
How to deal with upset patients (communication/counselling skills).	☐	☐	☐
Working with non-English language patients.	☐	☐	☐
How to support the participant to answer sensitive questions.	☐	☐	☐
How to collect Quality of Life/Patient-Reported Outcome data without biasing the results.	☐	☐	☐
Collecting Quality of Life/Patient-Reported Outcome data in different patient groups and/or settings.	☐	☐	☐
Relevance and reasoning behind individual Quality of Life/Patient-Reported Outcome questions.	☐	☐	☐
How to deal with difficult situations.	☐	☐	☐

Other(s) (please specify)

National Data Manager/Coordinator Survey

Introduction

Thank you for your interest in this research

What is the purpose of this study?

The aim of this national trials unit survey is to gather information on how data coordinators/inputters and data managers deal with quality of life and other patient-reported outcomes in clinical trials. We will donate £2 to Cancer Research UK for each completed survey we receive.

Who is doing this research?

This research is being conducted by the 'Patient-Reported Outcomes Research Group' based in Primary Care Clinical Sciences at the University of Birmingham. The study forms part of a PhD being undertaken by Derek Kyte MSc, supervised by Dr Melanie Calvert PhD, Professor Heather Draper PhD and Dr Jonathan Ives PhD. The West Midlands Research Ethics Committee have favorably reviewed the study (Reference number: 12/WM/0068).

How long will it take?

The questionnaire has 15 questions, expected completion time is 10-15 minutes.

How will my data be protected?

All of the data collected from you will be kept anonymous. You will not be asked for any personal information, such as your name, date of birth or contact details, any other potentially identifying data will be kept confidential or anonymised. We will ask you about your work experience, but this information will be analysed at a group level and individual details will not be shared. The results of the questionnaire will be securely stored on the computer systems in Primary Care Clinical Sciences at the University of Birmingham for the duration of 10 years. After this period they will be deleted so that they cannot be recovered. Reports derived from the questionnaire results will be published in peer reviewed scientific journals, all data will be anonymous.

Once I agree to take part, can I change my mind?

You can exit the questionnaire at any point prior to submission and your answers will not be analysed. Once you have submitted the completed questionnaire you will not be able to withdraw as there is no way we can retrieve your anonymised answers.

Who can I contact to ask any questions?

Mr Derek Kyte
Phone: 0121 4158502, Email: d.g.kyte@bham.ac.uk

Who can I contact if I wish to make a complaint?

Dr Melanie Calvert
Phone: 0121 4148595, Email: m.calvert@bham.ac.uk

Please note: by advancing to the next page, you are consenting to take part in the study. Anonymity and confidentially will be ensured.

Explanation

DEFINITIONS Of TERMS USED IN THE SURVEY

Patient-reported outcome measures

Patient-Reported Outcome measures ask the patient a series of questions in order to gauge their views on their own health or care, i.e. an outcome directly reported by the patient.

Quality of Life

A Quality of Life measure is a Patient-Reported Outcome which is designed to evaluate the way in which physical, emotional and social well-being are affected by a disease or its treatment.

SECTION 1

This section asks questions about your experience of the **last** clinical trial you worked on that used a quality of life or other patient-reported outcome measure. It does not matter if this was a primary or secondary outcome in the trial.

Please be aware that there are no 'right' or 'wrong' answers in this survey. We are simply interested in hearing about your experiences and opinions.

*1. Thinking about the last clinical trial you worked on that used a quality of life or other patent-reported outcome measure.

How would you describe your role?

- ○ Data manager
- ○ Data coordinator
- ○ Data inputter

Other (please specify)

2. How long have you worked as a [Q1], in total?

- ○ Less than 1 year
- ○ 1-3 years
- ○ 4-6 years
- ○ 7-9 years
- ○ 10 years or more

3. To which of the following age groups do you belong?

- ○ 25 or younger
- ○ 26-35
- ○ 36-45
- ○ 46-55
- ○ 56 or older

4. Thinking about the last trial you worked on that used a Quality of Life or other Patient-Reported Outcome measure.

Was the trial based in primary care or secondary care?

- ○ Primary Care
- ○ Secondary Care

Other (please specify)

5. Which of the following clinical areas did the trial cover? PLEASE TICK ALL THAT APPLY

- ☐ General Practice
- ☐ Orthopaedics
- ☐ General Medicine
- ☐ Rheumatology
- ☐ Cardiovascular
- ☐ Oncology
- ☐ Elderly Care
- ☐ Respiratory
- ☐ Opthalmology
- ☐ Obstetrics & Gynaecology
- ☐ Paediatrics
- ☐ Neurology
- ☐ DON'T KNOW

Other(s) (please specify)

6. Which of the following Patient-Reported Outcome Measures did the trial use? PLEASE TICK ALL THAT APPLY

- ☐ Euroqol EQ-5D
- ☐ Health Assessment Questionnaire (HAQ)
- ☐ Nottingham Health Profile (NHP)
- ☐ SF-12® Health Survey or SF-12v2™ Health Survey
- ☐ SF-36® Health Survey or SF-36v2™ Health Survey
- ☐ Hospital Anxiety and Depression scale (HAD)
- ☐ Arthritis Impact Measurement Scales (AIMS2)
- ☐ EORTC QLQ - C30 (Core Questionnaire)
- ☐ Minnesota Living with Heart Failure © Questionnaire (MLHF)
- ☐ Oxford Hip Score (OHS)
- ☐ Oxford Knee Score (OKS)
- ☐ Roland-Morris Disability Questionnaire (RMDQ)
- ☐ Don't know
- ☐ Can't remember

Other(s) (please specify)

SECTION 1 - Last trial

7. Thinking about the last trial you worked on that used a Quality of Life or other Patient-Reported Outcome measure.

When the Quality of Life/Patient-Reported Outcome questionnaire data were inputted, which of the following occurred? PLEASE TICK ALL THAT APPLY

- ☐ The questionnaire was checked to see if the participant had completed all questions.
- ☐ If items were found to be missing, trial participants were followed up in some way (e.g. by post, by phone or via their research nurse) in order to complete the questionnaire.
- ☐ The questionnaire was checked for scoring errors (e.g. two answers given instead of one, or reversed scoring).
- ☐ If scoring errors were detected, trial participants were followed up in some way (e.g. by post, by phone or via their research nurse) in order to correct them.

Other (please specify)

8. Again, thinking about the last trial you worked on that used a Quality of Life or other Patient-Reported Outcome measure.

Please read the following statements. In each case, please answer 'yes', 'no', 'don't know', or 'not applicable'.

	Yes	No	Don't Know	Not applicable
The trial **protocol** included information about Quality of Life/Patient-Reported Outcome data inputting.	◯	◯	◯	◯
I felt the trial **protocol** content covering Quality of Life/Patient-Reported Outcome data inputting was adequate.	◯	◯	◯	◯
I received **trial training** which included information on Quality of Life/Patient-Reported Outcome data inputting.	◯	◯	◯	◯
I felt the **trial training** I received covering Quality of Life/Patient-Reported Outcome data inputting was adequate.	◯	◯	◯	◯
I had access to a **standard operating procedure** which included information on Quality of Life/Patient-Reported Outcome data inputting.	◯	◯	◯	◯
I felt the **standard operating procedure** covering Quality of Life/Patient-Reported Outcome data inputting was adequate.	◯	◯	◯	◯

You may expand upon your answers here (optional)

SECTION 2 - General thoughts

SECTION 2

This section will ask about your **general** thoughts about working with Quality of Life/Patient-Reported Outcome Measures in trials.

SECTION 2 - General thoughts

9. Some data managers/inputters we have spoken to have reported encountering Quality of Life/Patient-Reported Outcome data which raised concern for the wellbeing of the trial participant in some way.

This information has been termed: 'concerning' Patient-Reported Outcome information.

In these reports, 'concerning' Patient-Reported Outcome information may have been extreme questionnaire scores, or sometimes a participant might have written additional information on the questionnaire which raised concern (or attached a letter).

Thinking about your <u>general</u> experience. Have you ever encountered any 'concerning' Patient-Reported Outcome information within a trial?

- No
- Yes
- Don't know

SECTION 2 - General thoughts

10. Have you ever taken action in response to 'concerning' Patient-Reported Outcome information you have encountered within a trial, in order to assist a trial participant?

- No
- Yes

You may provide details of the action taken here (optional)

11. Were you able to record <u>all</u> action(s) taken in response to viewing 'concerning' Patient-Reported Outcome information, in the trial documentation?

- No
- Yes
- Not applicable

SECTION 2 - General thoughts

12. If you were to encounter 'concerning' Patient-Reported Outcome information in a future trial, for example, evidence of anxiety or depression, which of the following might you consider doing? PLEASE TICK ALL THAT APPLY

- ☐ I would not intervene, there is nothing I could do.
- ☐ I would not intervene, it is the responsibility of the trial participant's GP and regular healthcare team to monitor and deal with quality of life related disorders such as anxiety and depression, not the trial staff.
- ☐ I would discuss the findings with my line manager in the trial, or with the PI.
- ☐ I would discuss the findings with the participants research nurse.
- ☐ I would discuss the findings with a data manager/inputter colleague.

Other (please specify)

13. Please read the following statements. In each case, please answer 'yes', 'no', or 'don't know'.

	Yes	No	Don't know
There is usually specific guidance on dealing with **'concerning'** Patient-Reported Outcome information contained in trial protocols.	○	○	○
I usually receive training on what to do if I encounter **'concerning'** Patient-Reported Outcome information.	○	○	○
I feel confident about dealing with **'concerning'** Patient-Reported Outcome trial information.	○	○	○

You may expand upon your answers here (optional)

SECTION 3 - The future

SECTION 3

This final section will ask about the changes you would like to see regarding Quality of Life/Patient-Reported Outcome measurement in **future trials**.

SECTION 3 - The future

14. Thinking about the future.

Please read the following statements. In each case, please indicate whether you 'strongly agree', 'agree', have 'no opinion', 'disagree' or 'strongly disagree' with the statement.

	Strongly Agree	Agree	No Opinion	Disagree	Strongly Disagree
There should be more **protocol content and trial training** for data managers/inputters, covering Quality of Life/Patient-Reported Outcome measurement.	○	○	○	○	○
There should be **site manuals or standard operating procedures** available to data mangers/inputters that include information on Quality of Life/Patient-Reported Outcome administration in the trial.	○	○	○	○	○
There should be specific protocol content and trial training for data managers/inputters on how to deal with **'concerning' Patient-Reported Outcome information** in trials.	○	○	○	○	○

You may expand upon your answers here (optional)

15. Thinking about the future.

In your opinion, what particular Quality of Life/Patient-Reported Outcome data collection guidance do you feel is essential in helping you do your job well, and should be included the trial protocol, what should be included in trial training, and what should be included in a standard operating procedure? PLEASE TICK ALL THAT APPLY

	Trial Protocol	Trial Training	Standard Operating Procedure
How to input Quality of Life/Patient-Reported Outcome data into the database.	☐	☐	☐
What to do if there is missing data.	☐	☐	☐
What to do in the event of scoring errors (e.g. two answers instead of one, or reversed scoring).	☐	☐	☐
What to do if participants write additional information on their questionnaires (or attach a letter).	☐	☐	☐
When action should be taken in the event of extreme questionnaire scores.	☐	☐	☐
Who to speak to if there is concern about a participants Quality of Life/Patient-Reported Outcome data.	☐	☐	☐

Other(s) (please specify)

National Trial Manager Survey

Introduction

Thank you for your interest in this research

What is the purpose of this study?

The aim of this national survey is to gather information on how trial managers coordinate quality of life and other patient-reported outcome measurement in clinical trials. We will donate £2 to Cancer Research UK for each completed survey we receive.

Who is doing this research?

This research is being conducted by the 'Patient-Reported Outcomes Research Group' based in Primary Care Clinical Sciences at the University of Birmingham. The study forms part of a PhD being undertaken by Derek Kyte MSc, supervised by Dr Melanie Calvert PhD, Professor Heather Draper PhD and Dr Jonathan Ives PhD. The West Midlands Research Ethics Committee have favorably reviewed the study (Reference number: 12/WM/0068).

How long will it take?

The questionnaire has 14 questions, expected completion time is less than 10 minutes.

How will my data be protected?

All of the data collected from you will be kept anonymous. You will not be asked for any personal information, such as your name, date of birth or contact details, any other potentially identifying data will be kept confidential or anonymised. We will ask you about your work experience, but this information will be analysed at a group level and individual details will not be shared. The results of the questionnaire and any reports derived from it will be securely stored on the computer systems in Primary Care Clinical Sciences at the University of Birmingham for the duration of 10 years. After this period they will be deleted so that they cannot be recovered. Reports derived from the questionnaire results will be published in peer reviewed scientific journals, all data will be anonymous.

Once I agree to take part, can I change my mind?

You can exit the questionnaire at any point prior to submission and your answers will not be analysed. Once you have submitted the completed questionnaire you will not be able to withdraw as there is no way we can retrieve your anonymised answers.

Who can I contact to ask any questions?

Mr Derek Kyte
Phone: 0121 4158502, Email: d.g.kyte@bham.ac.uk

Who can I contact if I wish to make a complaint?

Dr Melanie Calvert
Phone: 0121 4148595, Email: m.calvert@bham.ac.uk

Please note: by advancing to the next page, you are consenting to take part in the study. Anonymity and confidentially will be ensured.

Explanation

National Trial Manager Survey

DEFINITIONS OF TERMS USED IN THE SURVEY

Patient-reported outcome measures

Patient-Reported Outcome measures ask the patient a series of questions in order to gauge their views on their own health or care, i.e. an outcome directly reported by the patient.

Quality of Life

A Quality of Life measure is a Patient-Reported Outcome which is designed to evaluate the way in which physical, emotional and social well-being are affected by a disease or its treatment.

SECTION 1

The next section asks questions about your experience of the **last** clinical trial you worked on that used a quality of life or other patent-reported outcome measure. It does not matter is this was a primary or secondary outcome in the trial.

SECTION 1 - Last trial

1. First, some questions about yourself. How long in total have you worked as a trial manager?

- Less than 1 year
- 1-3 years
- 4-6 years
- 7-9 years
- 10 years or more

2. To which of the following age groups do you belong?

- 25 or younger
- 26-35
- 36-45
- 46-55
- 56 or older

3. Thinking about the last trial you worked on that used a Quality of Life or other Patient-Reported Outcome measure.

Which of the following clinical areas did the trial cover? PLEASE TICK ALL THAT APPLY

- ☐ General Practice
- ☐ Orthopaedics
- ☐ General Medicine
- ☐ Rheumatology
- ☐ Cardiovascular
- ☐ Oncology
- ☐ Elderly Care
- ☐ Respiratory
- ☐ Opthalmology
- ☐ Obstetrics & Gynaecology
- ☐ Paediatrics
- ☐ Neurology

Other(s) (please specify)

4. Was the trial based in primary or secondary care?

- ○ Primary Care
- ○ Secondary Care

Other (please specify)

5. Which of the following Patient-Reported Outcome Measures did the trial use? PLEASE TICK ALL THAT APPLY

- ☐ Euroqol EQ-5D
- ☐ Health Assessment Questionnaire (HAQ)
- ☐ Nottingham Health Profile (NHP)
- ☐ SF-12® Health Survey or SF-12v2™ Health Survey
- ☐ SF-36® Health Survey or SF-36v2™ Health Survey
- ☐ Hospital Anxiety and Depression scale (HAD)
- ☐ Arthritis Impact Measurement Scales (AIMS2)
- ☐ EORTC QLQ - C30 (Core Questionnaire)
- ☐ Minnesota Living with Heart Failure © Questionnaire (MLHF)
- ☐ Oxford Hip Score (OHS)
- ☐ Oxford Knee Score (OKS)
- ☐ Roland-Morris Disability Questionnaire (RMDQ)
- ☐ Don't know
- ☐ Can't remember

Other(s) (please specify)

6. During the trial, were the staff involved in data collection given instructions on how to administer the quality of life/patient-reported outcome questionnaire?

- ○ No
- ○ Yes

You may expand upon your answer here (optional)

Section 1 - Last trial

7. Again, thinking about the same trial. What particular information on Quality of Life/Patient-Reported Outcome measurement was given to the data collection staff? Please read the options below and in each case select either 'included in trial protocol, training or SOP', or 'not included'.

	Included in trial protocol, training or SOP	Not included
The purpose and/or Importance of Quality of Life/Patient-Reported Outcome data to the trial.	○	○
Relevance and reasoning behind individual Quality of Life/Patient-Reported Outcome questions.	○	○
When to administer the questionnaire (time points).	○	○
When to administer the questionnaire during the clinic appointment (before/during/after the consultation).	○	○
How much assistance to give the participant during questionnaire completion.	○	○
How to check for, and deal with, missing Quality of Life/Patient-Reported Outcome data.	○	○
How to deal with Quality of Life/Patient-Reported Outcome information that raises concern for the wellbeing of the trial participant (e.g. a questionnaire indicating severe anxiety or depression).	○	○
What to do if participants write additional information on their questionnaires (or attach a letter).	○	○

You may expand upon your answers here (optional)

SECTION 2 - General thoughts

SECTION 2

This section will ask about your **general** thoughts about working with Quality of Life/Patient-Reported Outcome Measures in trials.

SECTION 2 - General thoughts

8. Some research nurses/data mangers we have spoken to have reported encountering Quality of Life/Patient-Reported Outcome questionnaires containing answers which raise concern for the wellbeing of the trial participant in some way.

This information has been termed: 'concerning' Patient-Reported Outcome information.

In these reports, 'concerning' Patient-Reported Outcome information may simply have been particularly extreme questionnaire scores, or sometimes a participant might have written additional information on the questionnaire which raised concern (or attached a letter); finally, some research nurses reported becoming concerned by things that the trial participant said to them either during, or after, the completion of the questionnaire.

Have you ever encountered/been made aware of any 'concerning' Patient-Reported Outcome information within a trial?

- No
- Yes
- Don't know

SECTION 2 - General thoughts

9. Have you ever taken action in response to 'concerning' Patient-Reported Outcome information you have encountered/been made aware of within a trial, in order to assist a trial participant?

- No
- Yes

You may provide details about the action taken here (optional)

10. Was there a mechanism in place to record all action(s) taken in response to the 'concerning' Patient-Reported Outcome information, in the trial documentation?

- ○ No
- ○ Yes
- ○ Not applicable

You may expand upon your answer here (optional)

SECTION 2 - General thoughts

11. If your data collection staff were to encounter 'concerning' Patient-Reported Outcome information in a future trial, for example, evidence of anxiety or depression, which of the following would you expect them to do? PLEASE TICK ALL THAT APPLY

- ☐ Not to intervene, it is the responsibility of the trial participant's GP and regular healthcare team to monitor and deal with quality of life related disorders such as anxiety and depression, not the trial staff.
- ☐ To discuss the findings with their line manager in the trial, or with the PI.
- ☐ To discuss the findings with a colleague.
- ☐ To discuss the findings with the participant.
- ☐ Using their discretion, arrange an appointment with the patient's GP or other appropriate healthcare professional.

Other (please specify)

SECTION 3 - The future

SECTION 3

This final section will ask about the changes you would like to see regarding Quality of Life/Patient-Reported Outcome measurement in **future trials**.

SECTION 3 - The future

12. Thinking about the future.

Please read the following statements. In each case, please indicate whether you 'strongly agree', 'agree', 'have no opinion', 'disagree' or 'strongly disagree' with the statement.

	Strongly Agree	Agree	No Opinion	Disagree	Strongly Disagree
Data collection staff in trials need more information on Quality of Life/Patient-Reported Outcome measurement - in the trial protocol.	○	○	○	○	○
Data collection staff in trials need more information on Quality of Life/Patient-Reported Outcome measurement - in other trial documentation, such as SOPs.	○	○	○	○	○
Data collection staff in trials need more information on Quality of Life/Patient-Reported Outcome measurement - delivered in the form of trial training.	○	○	○	○	○
There should be specific protocol content and trial training for data collection staff on how to deal with **'concerning' Patient-Reported Outcome information**.	○	○	○	○	○
It is important to explain to data collection staff, the purpose and Importance of Quality of Life/Patient-Reported Outcome data to the trial.	○	○	○	○	○
It is important to explain to data collection staff, the relevance and reasoning behind individual Quality of Life/Patient-Reported Outcome questions.	○	○	○	○	○

You may expand upon your answers here (optional)

13. Thinking about the future.

What particular Quality of Life/Patient-Reported Outcome guidance should be included the trial protocol, what should be included in trial training, what should be included in a standard operating procedure, and what guidance should not be included in any of the above? PLEASE TICK ALL THAT APPLY

	Trial Protocol	Trial Training	Standard Operating Procedure	None
Purpose/Importance of Quality of Life/Patient-Reported Outcome data in trial.	☐	☐	☐	☐
How to administer the questionnaire.	☐	☐	☐	☐
When to administer the questionnaire.	☐	☐	☐	☐
When/how to deal with 'concerning' Quality of Life/Patient-Reported Outcome information.	☐	☐	☐	☐
What to do if participants write additional information on their questionnaires (or attach a letter).	☐	☐	☐	☐
Ethical issues associated with Quality of Life/Patient-Reported Outcome use.	☐	☐	☐	☐
How to deal with upset patients (communication/counselling skills).	☐	☐	☐	☐
Working with non-English language patients.	☐	☐	☐	☐
How to support the participant to answer sensitive questions.	☐	☐	☐	☐
How to collect Quality of Life/Patient-Reported Outcome data without biasing the results.	☐	☐	☐	☐
Collecting Quality of Life/Patient-Reported Outcome data in different patient groups and/or settings.	☐	☐	☐	☐
Relevance and reasoning behind individual Quality of Life/Patient-Reported Outcome questions.	☐	☐	☐	☐
How to deal with difficult situations.	☐	☐	☐	☐

Other(s) (please specify)

14. Has completing this questionnaire changed the way you will manage quality of life/patient-reported outcome measurement in future trials?

- No
- Yes

You may expand upon your answer here (optional)

National CTU CI/PI Survey

Introduction

Thank you for your interest in this research

What is the purpose of this study?

The aim of this national survey is to gather information on how CI's/PI's in trials coordinate quality of life and other patient-reported outcome measurement. We will donate £2 to Cancer Research UK for each completed survey we receive.

Who is doing this research?

This research is being conducted by the 'Patient-Reported Outcomes Research Group' based in Primary Care Clinical Sciences at the University of Birmingham. The study forms part of a PhD being undertaken by Derek Kyte MSc, supervised by Dr Melanie Calvert PhD, Professor Heather Draper PhD and Dr Jonathan Ives PhD. The West Midlands Research Ethics Committee have favorably reviewed the study (Reference number: 12/WM/0068).

How long will it take?

The questionnaire has 14 questions, expected completion time is less than 10 minutes.

How will my data be protected?

All of the data collected from you will be kept anonymous. You will not be asked for any personal information, such as your name, date of birth or contact details, any other potentially identifying data will be kept confidential or anonymised. We will ask you about your work experience, but this information will be analysed at a group level and individual details will not be shared. The results of the questionnaire and any reports derived from it will be securely stored on the computer systems in Primary Care Clinical Sciences at the University of Birmingham for the duration of 10 years. After this period they will be deleted so that they cannot be recovered. Reports derived from the questionnaire results will be published in peer reviewed scientific journals, all data will be anonymous.

Once I agree to take part, can I change my mind?

You can exit the questionnaire at any point prior to submission and your answers will not be analysed. Once you have submitted the completed questionnaire you will not be able to withdraw as there is no way we can retrieve your anonymised answers.

Who can I contact to ask any questions?

Mr Derek Kyte
Phone: 0121 4158502, Email: d.g.kyte@bham.ac.uk

Who can I contact if I wish to make a complaint?

Dr Melanie Calvert
Phone: 0121 4148595, Email: m.calvert@bham.ac.uk

Please note: by advancing to the next page, you are consenting to take part in the study. Anonymity and confidentially will be ensured.

Explanation

National CTU CI/PI Survey

DEFINITIONS OF TERMS USED IN THE SURVEY

Patient-reported outcome measures

Patient-Reported Outcome measures ask the patient a series of questions in order to gauge their views on their own health or care, i.e. an outcome directly reported by the patient.

Quality of Life

A Quality of Life measure is a Patient-Reported Outcome which is designed to evaluate the way in which physical, emotional and social well-being are affected by a disease or its treatment.

Chief Investigator

The Chief Investigator (CI) is defined as the lead investigator for a single site study, or in relation to a study conducted at more than one site, the investigator who takes primary responsibility for the conduct of the study across all sites.

Principal Investigator

The Principal Investigator (PI) is defined as the authorised health professional responsible for the conduct of that study at a study site, and if a team of authorised health professionals at a study site conducts the study, the Principal Investigator is the leader responsible for that team.

SECTION 1

The next section asks questions about your experience of the **last** clinical trial you worked on that used a quality of life or other patent-reported outcome measure. It does not matter is this was a primary or secondary outcome in the trial.

SECTION 1 - Last trial

1. First, some questions about yourself. How much experience in total have you had as a CI/PI?

- ○ Less than 1 year
- ○ 1-3 years
- ○ 4-6 years
- ○ 7-9 years
- ○ 10 years or more

2. Which of the following age groups do you belong to?

- ○ 25 or younger
- ○ 26-35
- ○ 36-45
- ○ 46-55
- ○ 56 or older

3. Thinking about the last trial you worked on that used a Quality of Life or other Patient-Reported Outcome measure.

Which of the following clinical areas did the trial cover? PLEASE TICK ALL THAT APPLY

- ☐ General Practice
- ☐ Orthopaedics
- ☐ General Medicine
- ☐ Rheumatology
- ☐ Cardiovascular
- ☐ Oncology
- ☐ Elderly Care
- ☐ Respiratory
- ☐ Opthalmology
- ☐ Obstetrics & Gynaecology
- ☐ Paediatrics
- ☐ Neurology

Other(s) (please specify)

4. Was the trial based in primary or secondary care?

- ○ Primary Care
- ○ Secondary Care

Other (please specify)

5. In the study, Were you a CI or PI?

- CI
- PI

Other (please specify)

6. Which of the following Patient-Reported Outcome Measures did the trial use? PLEASE TICK ALL THAT APPLY

- Euroqol EQ-5D
- Health Assessment Questionnaire (HAQ)
- Nottingham Health Profile (NHP)
- SF-12® Health Survey or SF-12v2™ Health Survey
- SF-36® Health Survey or SF-36v2™ Health Survey
- Hospital Anxiety and Depression scale (HAD)
- Arthritis Impact Measurement Scales (AIMS2)
- EORTC QLQ - C30 (Core Questionnaire)
- Minnesota Living with Heart Failure © Questionnaire (MLHF)
- Oxford Hip Score (OHS)
- Oxford Knee Score (OKS)
- Roland-Morris Disability Questionnaire (RMDQ)
- Don't know
- Can't remember

Other(s) (please specify)

SECTION 1 - Last trial

7. At what stage of the trial design phase was the quality of life/patient-reported outcome element first discussed?

- From the start
- Part way through the process
- At the end of the process

You may expand upon your answer here (optional)

8. Was a quality of life/patient-reported outcomes expert involved in the design phase of the trial?

No

Yes

You may expand upon your answer here (optional)

9. In your opinion, on a scale of 1 (not important at all) to 10 (extremely important), with what level of importance did the trial management group view the quality of life/patient reported outcome(s) within the trial?

1 (not important at all)	2	3	4	5	6	7	8	9	10 (extremely important)

You may expand upon your answer here (optional)

10. During the trial, were the staff involved in data collection given instructions on how to administer the quality of life/patient-reported outcome questionnaire?

No

Yes

You may expand upon your answer here (optional)

Section 1 - Last trial

11. Again, thinking about the same trial. What particular information on Quality of Life/Patient-Reported Outcome measurement was given to the data collection staff? Please read the option below and in each case select either 'included in trial protocol, training or SOP', or 'not included'.

	Included in trial protocol, training or SOP	Not included
The purpose and/or Importance of Quality of Life/Patient-Reported Outcome data to the trial.	○	○
Relevance and reasoning behind individual Quality of Life/Patient-Reported Outcome questions.	○	○
When to administer the questionnaire (time points).	○	○
When to administer the questionnaire during the clinic appointment (before/during/after the consultation).	○	○
How much assistance to give the participant during questionnaire completion.	○	○
How to check for, and deal with, missing Quality of Life/Patient-Reported Outcome data.	○	○
How to deal with Quality of Life/Patient-Reported Outcome information that raises concern for the wellbeing of the trial participant (e.g. a questionnaire indicating severe anxiety or depression).	○	○
What to do if participants write additional information on their questionnaires (or attach a letter).	○	○

You may expand upon your answers here (optional)

SECTION 2 - General thoughts

SECTION 2

This section will ask about your **general** thoughts about working with Quality of Life/Patient-Reported Outcome Measures in trials.

SECTION 2 - General thoughts

12. Some research nurses we have spoken to have reported encountering Quality of Life/Patient-Reported Outcome questionnaires containing answers which raise concern for the wellbeing of the trial participant in some way.

This information has been termed: 'concerning' Patient-Reported Outcome information.

In these reports, 'concerning' Patient-Reported Outcome information may simply have been particularly extreme questionnaire scores, or sometimes a participant might have written additional information on the questionnaire which raised concern (or attached a letter); finally, some nurses reported becoming concerned by things that the trial participant said to them either during, or after, the completion of the questionnaire.

Have you ever encountered/been made aware of any 'concerning' Patient-Reported Outcome information within a trial?

- No
- Yes
- Don't know

SECTION 2 - General thoughts

13. Have you ever taken action in response to 'concerning' Patient-Reported Outcome information you have encountered/been made aware of within a trial, in order to assist a trial participant?

- No
- Yes

You may provide details here (optional)

14. Was there a mechanism in place to record all action(s) taken in response to the 'concerning' Patient-Reported Outcome information, in the trial documentation?

- No
- Yes
- Not applicable

You may expand upon your answer here (optional)

SECTION 2 - General thoughts

15. If your data collection staff were to encounter 'concerning' Patient-Reported Outcome information in a future trial, for example, evidence of anxiety or depression, which of the following would you expect them to do? PLEASE TICK ALL THAT APPLY

- Not to intervene, it is the responsibility of the trial participant's GP and regular healthcare team to monitor and deal with quality of life related disorders such as anxiety and depression, not the trial staff.
- To discuss the findings with their line manager in the trial, or with the PI.
- To discuss the findings with a colleague.
- To discuss the findings with the participant.
- Using their discretion, arrange an appointment with the patient's GP or other appropriate healthcare professional.

Other (please specify)

SECTION 3 - The future

SECTION 3

This final section will ask about the changes you would like to see regarding Quality of Life/Patient-Reported Outcome measurement in **future trials**.

SECTION 3 - The future

16. Thinking about the future.

Please read the following statements. In each case, please indicate whether you 'strongly agree', 'agree', 'have no opinion', 'disagree' or 'strongly disagree' with the statement.

	Strongly Agree	Agree	No Opinion	Disagree	Strongly Disagree
Data collection staff in trials need more information on Quality of Life/Patient-Reported Outcome measurement - in the trial protocol.	○	○	○	○	○
Data collection staff in trials need more information on Quality of Life/Patient-Reported Outcome measurement - in other trial documentation, such as SOPs.	○	○	○	○	○
Data collection staff in trials need more information on Quality of Life/Patient-Reported Outcome measurement - delivered in the form of trial training.	○	○	○	○	○
There should be specific protocol content and trial training for data collection staff on how to deal with **'concerning' Patient-Reported Outcome information**.	○	○	○	○	○
It is important to explain to data collection staff, the purpose and Importance of Quality of Life/Patient-Reported Outcome data to the trial.	○	○	○	○	○
It is important to explain to data collection staff, the relevance and reasoning behind individual Quality of Life/Patient-Reported Outcome questions.	○	○	○	○	○

You may expand upon your answers here (optional)

17. Thinking about the future.

What particular Quality of Life/Patient-Reported Outcome guidance should be included the trial protocol, what should be included in trial training, what should be included in a standard operating procedure, and what guidance should not be included in any of the above? PLEASE TICK ALL THAT APPLY

	Trial Protocol	Trial Training	Standard Operating Procedure	None
Purpose/Importance of Quality of Life/Patient-Reported Outcome data in trial.	☐	☐	☐	☐
How to administer the questionnaire.	☐	☐	☐	☐
When to administer the questionnaire.	☐	☐	☐	☐
When/how to deal with 'concerning' Quality of Life/Patient-Reported Outcome information.	☐	☐	☐	☐
What to do if participants write additional information on their questionnaires (or attach a letter).	☐	☐	☐	☐
Ethical issues associated with Quality of Life/Patient-Reported Outcome use.	☐	☐	☐	☐
How to deal with upset patients (communication/counselling skills).	☐	☐	☐	☐
Working with non-English language patients.	☐	☐	☐	☐
How to support the participant to answer sensitive questions.	☐	☐	☐	☐
How to collect Quality of Life/Patient-Reported Outcome data without biasing the results.	☐	☐	☐	☐
Collecting Quality of Life/Patient-Reported Outcome data in different patient groups and/or settings.	☐	☐	☐	☐
Relevance and reasoning behind individual Quality of Life/Patient-Reported Outcome questions.	☐	☐	☐	☐
How to deal with difficult situations.	☐	☐	☐	☐

Other(s) (please specify)

18. Has completing this questionnaire changed the way you will manage quality of life/patient-reported outcome measurement in future trials?

- No
- Yes

You may expand upon your answer here (optional)
